# Case of Peritonitis

**Published:** 1815-12

**Authors:** R. N. Starr

**Affiliations:** Surgeon.


*For the London Medical and Physical Journal.*

*Case of Peritonitis;* by R. N. Starr, Esq. Surgeon.

A MEDICAL friend having requested I would accompany him to visit a patient of his labouring under Peritonitis, the termination of which disease is so generally fatal, I beg leave to send the case, together with the treatment pursued.

*Case.*—About eight o'clock on the morning of Saturday, the 30th of September last, the wife of Josiah Roe, of Barnsley, was seized with symptoms of peritonitis. She had been delivered the preceding day of her first child. The symptoms were cold shiverings, acute pain of the abdomen, which was considerably swelled and painful to the touch; thirst intolerable; pulse quick and hard; skin hot; tongue furred, with constant nausea and inclination to vomit. The lochial discharge had been small, and was not of the usual appearance. Bleeding was immediately employed to the extent of twenty-four ounces taken from the arm, and wet cold cloths directed to be constantly applied to the abdomen. The Sulphas Magnesia cum Pil. Rhœi was given every two hours with a view of opening the bowels. Upon seeing her at noon, the pain had been evidently relieved for three hours after the bleeding, after which it had returned with equal violence. The cold cloths were discontinued, and warm fomentations substituted, as she fancied the former aggravated the symptoms. As the bowels remained confined, the medicines were continued, with the addition of two grains of calomel to each dose, an enema was injected, which produced two evacuations.

On seeing her again in the evening, the pains had returned with a constant vomiting; finding the pulse still quick and hard, with considerable heat of skin, bleeding was again employed to the extent of sixteen ounces, and which again relieved her,—the blood exhibiting that coriaceous surface, the same as in the previous bleeding. She was ordered to persevere in the use of the purgatives, and two more enemas were repeated, but without producing any more evacuations; and, by way of relieving the vomiting, small quantities of cold water were frequently given.

On Sunday morning, October 1, she was much in the same state in every respect, the medicines were continued, and the enemas repeated; the bleeding was also repeated to the quantity of ten ounces, which again had the effect of relieving her, and the blood exhibited the same appearance as before. At noon, the bowels remained still confined, the enemas were repeated, and the medicines directed to be given as usual. On seeing her in the evening, she appeared much the same, and it was proposed to give her the following formula:

℞. Aloes & G. Assafœtida, āā ʒ ſs. Calomel ℈i. Ol. Amsi. gts. ij. Syr. q. 1. mass in Pil. xviij. divide.

Of these she took three, with an ounce of the saline mixture every two hours. Upon seeing her the following morning, October 2d, she was much relieved, the pills had operated frequently; the skin had become moist; tongue cleaner; pulse softer; she had also passed a considerable quantity of urine, and the abdomen became less tumid and painful. The vomiting had nearly left her. She was ordered to persevere in the use of the pills and mixture; and, as the pains seemed relieved, the fomentations were discontinued.

At noon, she seemed much easier, and, the bowels having been pretty freely opened, the pills were discontinued, and the mixture directed to be given alone. The lochial discharge had appeared, together with milk in the breasts. In the evening she appeared easier.

℞. Pulv. Ipecac. Comp. g. xv. Hor. somni sumend.

On the morning of October 3d, she was evidently better in every respect, and in consequence was considered as convalescent.

*Remarks.*

*Remarks.*—In this case we had all the symptoms of peritonitis; yet, from the known fatality of the disease, I have my doubts whether it was a real case of *puerperal fever.* The blood, when drawn, had all the appearance of the same as when the system is in a state of increased vascular action; and the other symptoms present, all tended to confirm the same opinion. With regard to cold water in obstinate vomiting, I have repeatedly given it in small quantities frequently with evident advantage: it seemed to be of singular service in this case, both in relieving the distressing thirst and inclination to vomit.

*Barnsley;*
*October* 9, 1815.

